# Dances of death: macabre mirrors of an unequal society

**DOI:** 10.1007/s00038-012-0381-x

**Published:** 2012-06-15

**Authors:** Johan Pieter Mackenbach, Rolf Paul Dreier

**Affiliations:** Department of Public Health, Erasmus MC, Rotterdam, The Netherlands

**Keywords:** History, Mortality, Social inequality, Art

## Abstract

**Objectives:**

Between 1400 and 1800, Dances of Death were a popular art form depicting a metaphorical encounter between Death and representatives of a stratified human society. We review the thematic development of Dances of Death and study the development of social critique.

**Methods:**

We first assembled a full catalogue of all Dances of Death created between 1400 and 1800. We then analyzed patterns of spatiotemporal diffusion and made an in-depth hermeneutic study of the combined texts and images of a carefully selected set of 20 Dances of Death, comparing four distinct periods (1425–1525, 1525–1600, 1600–1650, and 1650–1800).

**Results:**

We identified more than 500 Dances of Death. It was only in its first stage of development, coinciding with the Pre-Reformation (1425–1525), that social critique was very prominent. This was represented in four forms: explicit references to social (in) equality, to failures of the authorities, and to emancipated farmers, and a general social realism. In later phases social critique largely disappeared and was replaced by religious themes.

**Conclusions:**

Dances of Death provide historical context to current analyses and debates of social inequalities in health. They remind us of the stubbornness of these inequalities, which despite progress in material well-being are still very much with us today.

## Introduction

We live in an unequal world, with large variations between rich and poor in the likelihood of a long life. In Western Europe, inequalities in life expectancy at birth between those with a low and a high socioeconomic position amount to between 5 and 10 years (Commission on Social Determinants of Health 2008; Mackenbach et al. 2008). It is not exactly known when these inequalities in mortality originated, but parish registers from the seventeenth and eighteenth centuries show that substantial differences in mortality rates between persons with higher and lower social ranks were already present then (Perrenoud 1975; Schultz 1991). It is not certain whether socioeconomic inequalities in mortality also existed before the seventeenth or eighteenth century, but anecdotal evidence suggests that even mortality from the bubonic plague was higher in the lower social classes, partly because the very rich had the opportunity to escape from plague-ridden towns by going to their country houses, thereby reducing their risks of infection (Cipolla and Zanetti 1972).

**Fig. 1:**
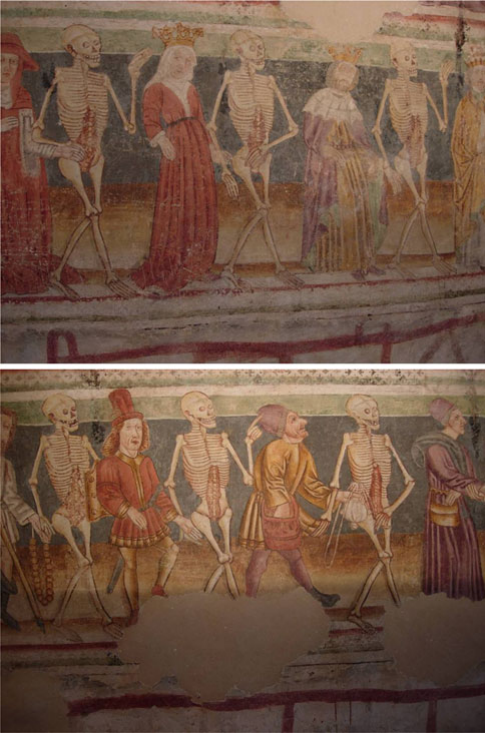
An example of a monumental Dance of Death: two fragments from the church of the Trinity, Hrastovlje (Slovenia). Source: photographs made by the authors

The ravages of plague epidemics caused great fear and contributed significantly to the rise of a whole series of new and macabre art forms, including Triumphs of Death (large paintings in which a personification of Death reaps masses of people), *Artes Moriendi* (illustrated treatises on how to die properly) and so-called Dances of Death (Huizinga 1924; Kurtz 1934; Clark 1950; Meiss 1951; Ariès 1977). It is commonly thought that the creation of Dances of Death followed the spread of the plague (Brossolet 1968; Schadewaldt 1991; Corvisier 1999). Dances of Death were artistic expressions of human mortality, executed either monumentally (mainly as a wall painting) or graphically (in the form of prints or books). They depict a metaphorical encounter between Death and representatives of human society in their last moments of earthly life. Death invites the dying to his dance and at the same time addresses them about their personal sins (Fig. 1). The basis of this *Memento mori* is the Christian idea of the spiritual equality of all mankind before Death. Paradoxically, to convey this message the backbone of the iconography of the Dance of Death is a representation of social *in*equality, the so-called *Ständereihe*, i.e. a depiction of the rigidly vertical social hierarchy which was believed to be ordained by God (Oexle 1988). Starting with Pope and Emperor all social positions would be represented, through Bishop and Squire, Usurer and Physician, all the way down to Clerk and Hermit, Jew and Turk. Each of these social positions was attributed its own typical sins, which were often sharply criticized. As a result, and because of the overrepresentation of the upper social classes, some of these Dances of Death read like early forms of social satire (Corvisier 1969; Werner 1975; Mackenbach 1995).

The question whether Dances of Death were really socially critical has attracted a lot of attention but has never been systematically analyzed. It is widely agreed that Dances of Death did not have ‘revolutionary’ intentions (Egger 1990), but did have a potential for social critique (Schulte 1991; Kiening 2003; Oosterwijk 2006). No studies, however, have been done of how this worked, who and what was criticized, and what the reasons were. The only exception is Petersmann (1983) who confined himself to one Dance of Death. Most of the existing literature on Dances of Death has been preoccupied with the origin and the various forms of appearance of the genre. Researchers from related fields also have only rarely included Dances of Death in their studies of ‘satire’ or ‘social critique’ (Mezger 1991).

This paper therefore aims to provide an analysis of the thematic development of Dances of Death in the period between 1400 and 1800, focusing on the theme of social critique (i.e., criticism of the structure of society and the inequalities it generates). In doing so, we build on a monograph written by one of us (Dreier 2010) and present some of the results of that historical study in a broader public health context. Our study period starts with the earliest Dances of Death and ends with the French Revolution when modern notions about social equality took root. We start by describing the spatiotemporal development of Dances of Death, and then show that it is mainly in its first stage of development, coinciding with the Pre-Reformation, and that critique of the existing social order was prominent.

## Methods

We first assembled a full catalogue of all Dances of Death created between 1400 and 1800. We included all Dances of Death mentioned in existing monographs and catalogues (e.g. Rosenfeld 1954; Hammerstein 1980; Utzinger and Utzinger 1996; Sörries 1998) and searched for hitherto unknown Dances of Death in libraries and library catalogues and during study trips in Germany, Switzerland, and Italy. For each Dance of Death we collected available information on current status, date of production, place of production, author or artist, language, composition of the ‘Ständereihe’, and state of preservation and accessibility. This comprehensive inventory of (currently) more than 500 Dances of Death has been placed online (http:\\www.totentanz.nl) and is further discussed in Dreier (2010).

We then studied the thematic development of these Dances of Death, by hermeneutic analysis of the combined texts and images of a carefully selected set of 20 Dances of Death. For this part of the study we selected Dances of Death for which full documentation (i.e., complete texts and images, and information on circumstances of production) was available, and which on the basis of patterns of spatiotemporal diffusion and artistic kinship were judged to represent important steps in the development of the genre. The analytic methods are further documented in Dreier (2010).

In our analyses we compared four periods, chosen to reflect distinct phases in the religious and political development of Europe: the period preceding the Reformation (1425–1525), the period of the Reformation (1525–1600), the period of the Counter-Reformation and Wars of Religion (1600–1650), and the period between the end of the Wars of Religion, and the French Revolution (1650–1800).

## Results

### Spatiotemporal development of Dances of Death (1400–1800)

**Fig. 2:**
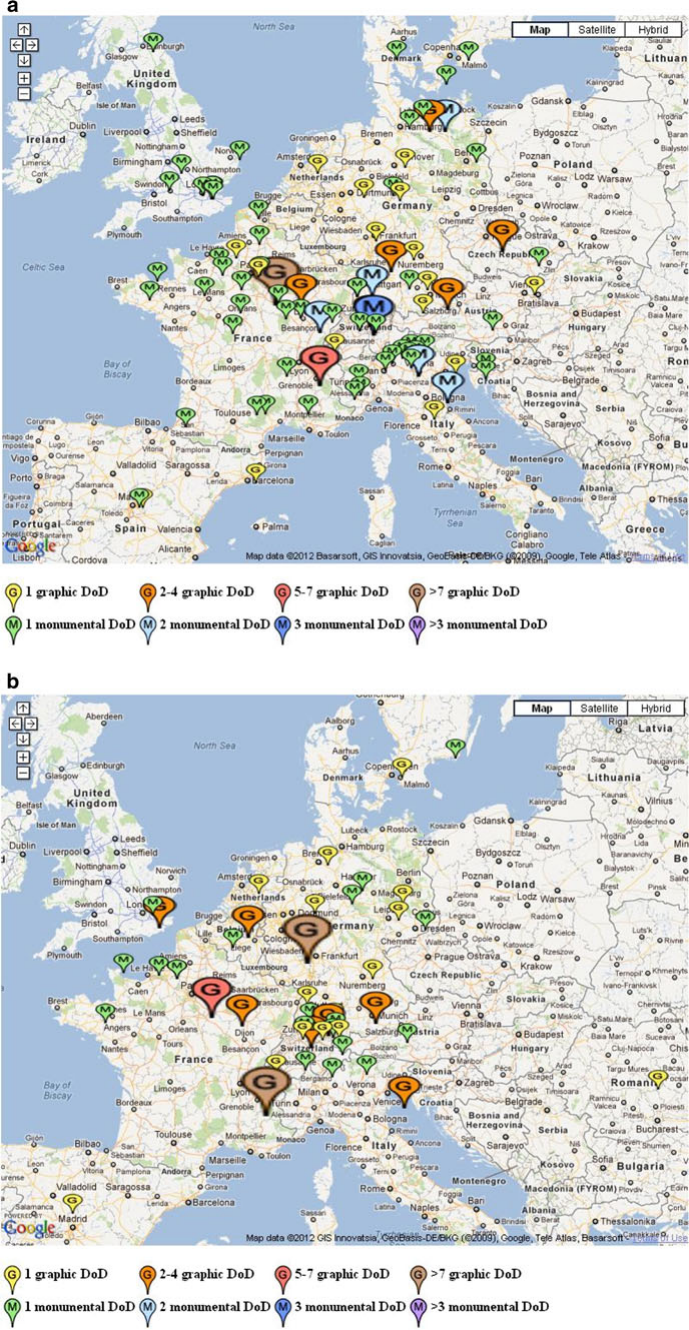

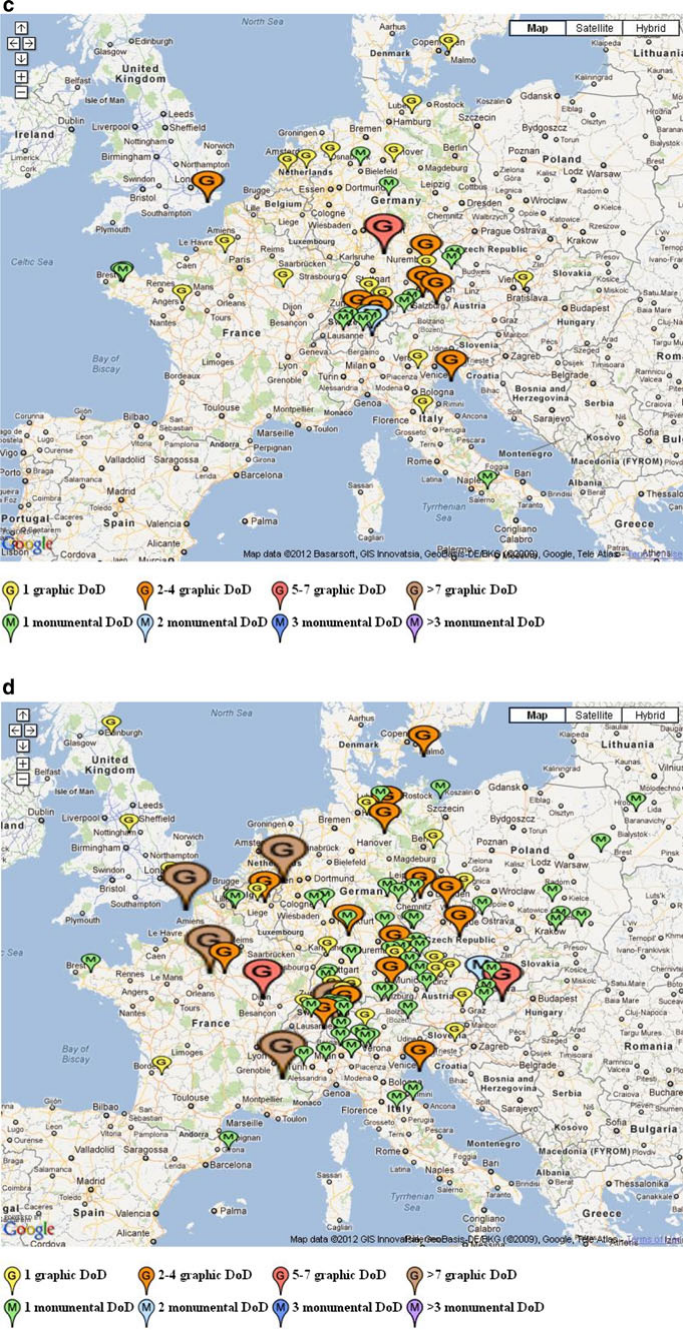
Geographical distribution of Dances of Death made between 1400 and 1800. *Small balloons* indicate single or small numbers of Dances of Death made in one place. The *largest balloons* indicate centers of production, where more than five Dances of Death were produced. **a** Geographical distribution of Dances of Death made between 1425 and 1525. **b** Geographical distribution of Dances of Death made between 1525 and 1600. **c** Geographical distribution of Dances of Death made between 1600 and 1659. **d** Geographical distribution of Dances of Death made between 1650 and 1800

We traced more than 500 Dances of Death created between 1400 and 1800, of which about one-third were monumental, and two-thirds were graphical works. Almost half of these Dances of Death were produced in the German-speaking lands of middle Europe (Fig. 2). There are very few Dances of Death east of the imaginary line running from Stockholm to Zagreb, which indicates that this art motive belongs to the Roman-Christian cultural space, as opposed to the Orthodox cultural space. Yet while France hosts a substantive number of *Danses Macabres*, important parts of the Roman-Christian cultural space, like the Iberian and Italian peninsulas, have only a small number, and there are also a few regions that are even absolutely Dance of Death-free, such as Ireland. Because Italy, Spain, and Ireland were hit very hard and repeatedly by bubonic plague throughout the centuries, the near-absence of Dances of Death in these countries shows that the geographical diffusion of this art form fails to match the geographical pathology of the epidemic. This indicates that the popular hypothesis that Dances of Death were a companion of the plague is incorrect.

**Fig. 3:**
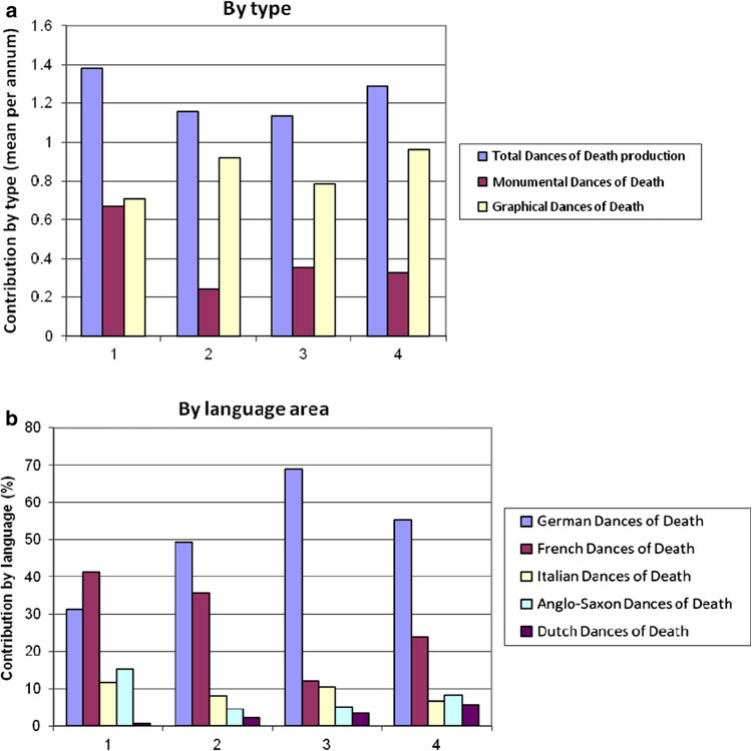
Temporal distribution of Dances of Death by type and language area. **a** Temporal distribution of Dances of Death by type. 1 = 1425–1525, 2 = 1525–1600, 3 = 1600–1650, 4 = 1650–1800. **b** Temporal distribution of Dances of Death by language area. 1 = 1425–1525, 2 = 1525–1600, 3 = 1600–1650, 4 = 1650–1800

When we relate the development and diffusion of this art motive to the four periods mentioned above, the following can be observed (Fig. 3). In the first or Pre-Reformation period (ca. 1425—ca. 1525) the Dance of Death spread rapidly—also thanks to the invention of print—from its breeding grounds in the north of France and the south of Germany, to reach its largest geographical distribution ever, including areas as far away as Scotland, Finland, Istria, and Andalusia. Although most Dances of Death were still made in France, where the first monumental Dance of Death had been painted in 1424, German-speaking areas also had a large share. Monumental and graphical Dances of Death had roughly equal shares in the total production. In this period, many Dances of Death were commissioned by preaching friars, such as the Dominicans and Franciscans, for whom these macabre pictures were illustrated sermons in which believers were urged to contemplate their sins and better their lives before it was too late. As a matter of fact, many of these Dances of Death included a preacher at the start and/or end of the “dance”. Dominicans and Franciscans strived for a reform of Catholic faith, in which greater emphasis was placed on the strict observance of biblical precepts, such as the rejection of material possessions. They advocated a new spirituality founded on the apostolic ideal of poverty, and they were critical of both religious and worldly leaders (Angenendt 1997). This ‘Pre-Reformation type’ of Dance of Death culminated in Holbein’s *Bilder des Todes* (created in 1524/25, published in 1538), an immensely popular book illustrated with wood-cuts providing the muster for many Dances of Death in the following period (Holbein 1971).

In the second period (ca. 1525–ca. 1600) the center of production gradually started to shift to German-speaking areas (present-day Germany, Switzerland, North of Italy). This phase coincided with a very divisive period of church history, ending in a definitive split between the Roman-Catholic church and various Protestant denominations. The Reformation also had a profound influence on the character of Dances of Death and gave rise to a ‘Reformation type’: Dances of Death now became vehicles of theological dispute between Roman Catholicism and reformed Lutheranism or Calvinism. A large majority of Dances of Death produced in this period were commissioned by Protestants and therefore contained sharp criticisms of the Catholic clergy and doctrine. This largely replaced the broader, egalitarian critique of the previous phase. There was a rapid increase in the share of graphical Dances of Death: prints and books provided a convenient means of transportation of the potentially subversive message. Holbein’s prints were re-used many times, sometimes with small variations, but the texts accompanying the pictures were rewritten to make them more explicitly polemical towards Catholicism.

In the beginning of the seventeenth century, Roman-Catholics rediscovered the Dance-of-Death motif and adapted it ingeniously to their own needs. In this third period (ca. 1600–ca. 1650), the rate with which new Dances of Death were produced was a little lower as compared with the previous phase, probably due to the Wars of Religion, such as the Thirty Years’ War (1618–1648) which was fought in Germany at this time. The focus of production now clearly lay in German-speaking areas, particularly in the southern half of present-day Germany and neighboring areas in Switzerland and Austria. Most of the Dances of Death produced in this third phase were commissioned by Roman-Catholics, and focused on the traditional *Memento Mori*. Critique of churchly or world leaders was largely absent in these Dances of Death. Although the Jesuits did not directly commission them, the similarity between the content of these Catholic Dances of Death and Jesuit theology and didactic (Schwaiger 1998) suggests their involvement in the development and diffusion of this ‘Counter-Reformation type’ of Dances of Death.

In the fourth period (ca. 1650–ca. 1800) many new Dances of Death were created, exceeding the rate with which they were produced in the two previous phases. The focus of production stayed in German-speaking areas, with some further expansion towards the East, probably following German colonization of present-day Poland and Byelorussia. The Catholic Dance of Death slowly evolved further on the basis developed in the previous phase, without important thematic developments. Many Dances of Death repeated the well-known examples for rural and often remote parishes. Protestant Dances of Death almost disappeared altogether. It is likely that the diminishing popularity of the Dance-of-Death motive among Protestants was due to its confrontational style which became increasingly outdated towards the end of the Thirty Years’ War. This was a historical period characterized by a cautious appeasement between the Christian confessions (Schulze 1998).

### The rise and fall of social critique in Dances of Death

Our in-depth analysis of the thematic development of Dances of Death shows that social critique, in the form of social satire with egalitarian undertones, was largely limited to the first phase (1425–1525). In this Pre-Reformation phase, four elements of the text and/or images of the Dances of Death contributed to the overall message of social critique: explicit references to social (in)equality, representations of the failures of the authorities, representations of emancipated farmers, and a general social realism in all representations.

**Fig. 4:**
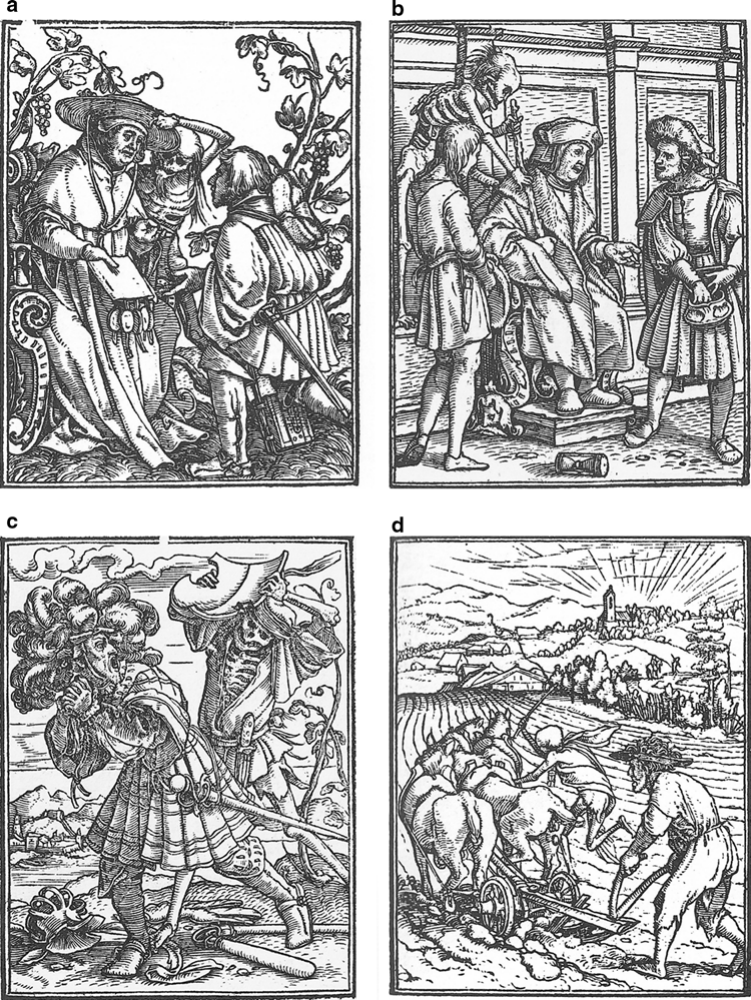
Examples of Holbein’s prints. Cardinal, Judge, Nobleman with farmer, Farmer. **a** Example of Holbein’s prints: Cardinal. **b** Example of Holbein’s prints: Judge. **c** Example of Holbein’s prints: Nobleman with farmer. **d** Example of Holbein’s prints: Farmer. Source: Holbein (1971). The Dance of Death. Facsimile edition. Dover, New York. Original edition published in Lyon in 1538

While the earliest Dances of Death only argued that all men are equal *in* death, i.e. face the same fate in the afterlife, most Pre-Reformation Dances of Death carried the notion that all men are equal *before* death, in a very concrete sense. One Dance of Death states: “Therefore understand this all/we must all go into the earth/and let nobody raise himself because of his nobility or power/his riches or his fine appearance” [Knoblochtzer H. Der doten dantz mit figuren, clage und antwort schon von allen staten der werlt. UB Heidelberg, GW M 47 257, 22 recto (ca. 1488)]. And another one: “Oh you child of man, come here/and look to see who is the lord and who the servant/because God does justice according to the law/here lies the lord and the servant too” [Monumentaltotentanz von Kientzheim. Reproduction in Stehle B. Der Totentanz von Kienztheim im Ober-Elsass. Strassburg: 1899, 24]. The idea that the principle of biblical equality of all men is also valid for the visible world is apparent in frequent representations of courts of justice, which show biased kings and judges *not* to treat everyone equally according to his right. Holbein’s image of the judge shows him to be corrupt and evil, but Death stops him from making a verdict favoring the rich (Fig. 4).

Pre-Reformation Dances of Death also are full of the visible failures of authorities, both religious and secular. A desire of the authors for church and political reform can often easily be discerned. In Knoblochtzer’s Dance of Death, for example, the bishop accuses himself with the following words: “I have received a lot of money/and repressed the poor with violence” [Knoblochtzer H. Der doten dantz mit figuren, clage und antwort schon von allen staten der werlt. UB Heidelberg, GW M 47 257, 3 verso]. Holbein shows the cardinal receiving money from a rich nobleman, in return for a document with seals, but Death lifts his hat to show that he will lose his authority (Fig. 4). Princes, noblemen and urban authorities were also often attacked vehemently for neglecting their duties and for their lack of righteousness. In the earliest Dances of Death these worldly authorities were only represented as abstract representatives of the higher order, but towards the end of the fifteenth century Dances of Death criticize their way of life and rule in very concrete ways. In the Kientzheim Dance of Death, Death says of the emperor: “Widows, orphans and land and people/I have not kept in peace./I laid siege, waged a lot of war/and rarely considered public welfare” [Monumentaltotentanz von Kientzheim. Reproduction in Stehle B. Der Totentanz von Kienztheim im Ober-Elsass. Strassburg: 1899, 28–29].

In the earliest Dances of Death, farmers represented the rather abstract ideal of the biblical Adam: diligence, austerity, and humble acceptance of poverty. But gradually the representation of farmers changed: they became defensible and self-confident. Farmers now could say: “Large works, chopping, plowing/I did at all times/to nourish wife and child/I never wasted money/I already paid my tithes/Serving God and going to church/I never failed out of misery/Therefore death falls me lighter” [Monumentaltotentanz von Kientzheim. Reproduction in Stehle B. Der Totentanz von Kienztheim im Ober-Elsass. Strassburg: 1899, 51]. Holbein has two farmer’s scenes: one according to the traditional scheme, in which Death relieves the hard working plowman, and one in which Death is disguised as a fighting farmer attacking a nobleman (Fig. 4). The flail on the ground provides a direct reference to the *Bauernkrieg*, the German peasant’s revolt of 1524/25, and Death’s taking side with the farmers in that conflict is undisputable.

More generally, Pre-Reformation Dances of Death slowly but increasingly took the form of a concrete social portrait. The typical sins of each social position were no longer represented in an abstract way, but more specifically, often referring to local circumstances. In one Dance of Death from a famous wine-growing region the usurer says: “I purchase wine and grain/I am delighted when it becomes expensive” [Monumentaltotentanz von Kientzheim. Reproduction in Stehle B. Der Totentanz von Kienztheim im Ober-Elsass. Strassburg: 1899, 48]. Unequal chances of social mobility are referred to when another Dance of Death lets the merchant say: “All nights and days I must wake/in order to make my children into lords/my heart was possessed by earthly goods/when I have to leave I will have a lot of pain” [Monumental Dance of Death of Bern. Reproduction in Tripps J. Den Würmern wirst du Wildbret sein. Der Berner Totentanz des Niklaus Manuel Deutsch in den Aquarellkopien von Albrecht Kauw. Bern: 2005, 75].

In Pre-Reformation Dances of Death critique of the servants of the faith and critique of inequality went hand in hand, because both served the intimately linked religious and political agendas of the Dominicans and Franciscans and other religious reform movements. But when the Reformation took off, in the early decades of the sixteenth century, Dances of Death mainly became vehicles for confessional strife. Egalitarian critique did not immediately disappear, as is clear from some of Gilles Corrozet’s text variations accompanying Holbein’s pictures: “The lower classes will revolt/against inhumanity/and will take the violent/with them without effort” [Holbein H. The Dance of Death. Facsimile edition. New York, NY: Dover, 1971, 24]. But gradually these Protestant Dances of Death became dominated by church polemics: “You Pope, a real antichrist/wants to be the earthly God/you are only a mendacious man/must now be mocked/in order for the world to see/whom it has honored/you do not love God, only splendor and money/it has lasted long enough” [Denecker J. Todtentantz. Augsburg: 1544, n.p].

References to social equality and inequality became rare, and references to injustice were rephrased to remove the sharper, socio-politically subversive edges. During the sixteenth century, Dances of Death seemed to have lost their appetite for a broader social reform, just like Luther condemned the social unrest of the German Peasant’s revolt in favor of social order (Briggs and Burke 2005). Echoing Luther’s respect for secular authority, a Protestant Dance of Death lets the emperor say: “The upper leader, the only lord/of the whole world I am/for whom stretches nobility and honor/and a lot of grace./Here on earth the highest position/I have in my power/the empire, and also much land of my own/which I keep in peace” [Denecker J. Todtentantz. Augsburg: 1544, n.p]. The contrast with earlier representations of the emperor could not have been more striking.

## Discussion

Even though criticism of social inequality, or of the higher social classes, has never been the main theme of Dances of Death, criticism targeting the behavior of people in the visible world was an important part of the message. The *Memento Mori* of the Dance of Death was directed against all members of society, and the original intention of including a *Ständereihe* was probably just to represent the whole of society. But the *Memento Mori* also required that the common failures of each social position would be enumerated, and because the number of different higher social positions happened to be larger than that of lower social positions, Dances of Death could easily be perceived as criticizing the higher social classes more than ordinary folk.

This effect was strengthened by the fact that most monumental Dances of Death were to be found in public places, e.g. not only inside churches but also on their outer walls, and therefore publicly visible, and easily accessible. Texts were often simple rhymes and commonly written in the vernacular. Highly placed persons sometimes represented real living or historical persons whom the local population would recognize. This was all part of the public preaching strategy of the Dominicans and Franciscans, whose mission was to convert the less privileged members of society (Schwaiger 1998). As a result, Dances of Death came to resemble a form of public chastisement of the authorities, which in its turn probably contributed to their popularity.

Also, the paradoxical nature of Dances of Death, in which the inequality of the *Ständereihe* was used to explain that all human beings are equal before death, created an inherent tension which must have been obvious to everyone, and which almost invited the spectators to reflect on the legitimacy of a strictly hierarchical society. Medieval theology had argued that the *Ständereihe* represented God’s design of society, in which all members, humble and high, were equally valuable parts of the same body (Heineman 1966). This conservative ideal, however, was undermined by the sharp criticism of the higher social positions. That all ranks of society danced together further contributed to the paradoxical nature of Dances of Death: such a common dance representing the equality of all mankind was the ‘world upside down’ (Schindler 2002).

In the late Middle Ages and early Modern period, people in Europe seem to have been unaware of social inequalities in life expectancy, and even if they would have known that those with higher social positions could evade death a bit longer than the poor, they would probably not have cared. They were more interested in the afterlife, and for them the bitter inequalities during life on earth could, to some extent, be traded off against the equality of life in heaven.

But they were very much aware of social inequalities in access to material and immaterial resources. During one particular period, ca. 1425–ca. 1525, this awareness gave rise to public and concrete criticism of various aspects of social inequality within the framework of a popular religious art form, the Dance of Death. This critique was sanctioned by parts of the Church, as the underpinnings of a social reform movement which later dissolved in the theological struggles of the Reformation and Counter-Reformation.

Although many of these Dances of Death have not survived the passage of time, sufficient numbers remain to remind us of the long history of social critique—and the stubbornness of social inequality, which despite enormous progress in material well-being is still very much with us today, despite the fact that we now know it to be strongly associated with inequality in the length of life (Mackenbach 1996). Dances of Death, therefore, provide historical context to current analyses and debates of social inequalities in health.
